# Regional SUV quantification in hybrid PET/MR, a comparison of two atlas-based automatic brain segmentation methods

**DOI:** 10.1186/s13550-020-00648-8

**Published:** 2020-06-08

**Authors:** Weiwei Ruan, Xun Sun, Xuehan Hu, Fang Liu, Fan Hu, Jinxia Guo, Yongxue Zhang, Xiaoli Lan

**Affiliations:** 1 Department of Nuclear Medicine, Union Hospital, Tongji Medical College, Huazhong University of Science and Technology, No. 1277 Jiefang Ave, Wuhan, 430022 China; 2 Hubei Province Key Laboratory of Molecular Imaging, Wuhan, 430022 China; 3 GE Healthcare, Shanghai, 201203 China

**Keywords:** PET/MR, Segmentation, Atlas-based, Parkinson disease, Multiple system atrophy

## Abstract

**Background:**

Quantitative analysis of brain positron-emission tomography (PET) depends on structural segmentation, which can be time-consuming and operator-dependent when performed manually. Previous automatic segmentation usually registered subjects’ images onto an atlas template (defined as RSIAT here) for group analysis, which changed the individuals’ images and probably affected regional PET segmentation. In contrast, we could register atlas template to subjects’ images (RATSI), which created an individual atlas template and may be more accurate for PET segmentation. We segmented two representative brain areas in twenty Parkinson disease (PD) and eight multiple system atrophy (MSA) patients performed in hybrid positron-emission tomography/magnetic resonance imaging (PET/MR). The segmentation accuracy was evaluated using the Dice coefficient (DC) and Hausdorff distance (HD), and the standardized uptake value (SUV) measurements of these two automatic segmentation methods were compared, using manual segmentation as a reference.

**Results:**

The DC of RATSI increased, and the HD decreased significantly (*P* < 0.05) compared with the RSIAT in PD, while the results of one-way analysis of variance (ANOVA) found no significant differences in the SUV_mean_ and SUV_max_ among the two automatic and the manual segmentation methods. Further, RATSI was used to compare regional differences in cerebral metabolism pattern between PD and MSA patients. The SUV_mean_ in the segmented cerebellar gray matter for the MSA group was significantly lower compared with the PD group (*P* < 0.05), which is consistent with previous reports.

**Conclusion:**

The RATSI was more accurate for the caudate nucleus and putamen automatic segmentation and can be used for regional PET analysis in hybrid PET/MR.

## Background

Positron-emission tomography (PET) is a molecular imaging method that uses the annihilation reactions of different positron emitters in radiotracers to generate images from the 511 keV gamma rays emitted. The positron emitters used, such as ^18^F and ^11^C, are bound to physiologically active substances to detect disease. Because physiologic images are relatively low in spatial resolution, computed tomography (CT) has been combined with PET for both anatomical location and attenuation correction. PET/CT scanning is widely applied in the evaluating of tumors, cardiac disease, CNS disorders, and infection/inflammation [1, 2].

Compared with CT, magnetic resonance imaging (MRI) has better soft tissue contrast and can obtain multiparametric images, e.g., T_1_-weighted images (T_1_WI), T_2_-weighted images (T_2_WI), proton density-weighted images (PDWI), or diffusion-weighted imaging (DWI), without ionizing radiation exposure. Therefore, the combination of PET and MRI should provide much more functional and structural information than CT without CT’s contribution to the overall radiation dose [3–5]. Combining PET with MRI was initially considered in the 1990s [6, 7]. Until 2010, with the advent of a magnet-compatible avalanche photodiode detector (APD), the first commercial whole-body hybrid PET/MR system (Siemens Biograph MRI scanner, Siemens Healthcare, Erlangen, Germany) [8] was introduced. In 2014, the latest generation of clinical whole-body hybrid PET/MR scanner (SIGNA PET/MR, GE Healthcare, Waukesha WI, USA) emerged with silicon photomultipliers (SiPMs), which digitize and process the signal directly within the magnetic field, which resulted in a thousand-fold improvement in time resolution, permitting time-of-flight (TOF) imaging [9].

PET/MR can simultaneously obtain images from the two modalities and is very useful for applications in neuroimaging. Catana et al. summarized the potential clinical application of PET/MR in patients with neurological disorders [10]. Previous studies have shown that the metabolic patterns of Parkinson’s disease (PD) and atypical Parkinson’s syndrome, e.g., multiple system atrophy (MSA), progressive supranuclear palsy (PSP), and corticobasal degeneration (CBD), were different [11]. However, it is usually difficult to quantitatively analyze abnormalities in different brain regions accurately [12]. Because of PET’s relatively low spatial resolution and the complexities of brain anatomy, the analysis of regional PET quantification relies on MRI. Brain structural segmentation based on MRI is very useful to localize regional CNS metabolism for clinical diagnosis.

Segmentation is one of the fundamental challenges in biomedical image analysis in that brain morphological characteristics are very complicated. This has been widely investigated to help diagnosis or surgery, e.g., deep brain stimulation (DBS) [13, 14]. Brain structural segmentation can be performed manually or automatically. Manual segmentation is highly time-consuming, requires expert anatomical knowledge [15, 16], and is subject to operator-dependence, especially when the signal-to-noise ratio or resolution is suboptimal. However, manual segmentation is still often employed, and the results are always used to establish a valid ground truth against which to assess automated segmentation results [17]. Automatic segmentation methods depending on algorithms can be very convenient, and the results are usually objective and reproducible [17]. Automatic segmentation methods must show accurate imaging co-registration, including the co-registration of images from different modalities, and accurate co-registration with a common reference template, such as the Montreal Neurological Institute (MNI) brain template [18].

For PET quantitative analysis, previous studies usually extracted the regional cerebral standardized uptake values (SUVs) directly based on an atlas, e.g., the widely used 3D stereotactic surface projection (3D SSP), or acquired the PET/CT and MRI data sequentially, registered the PET and MRI by postprocessing [19–21], and performed quantitative SUV analysis. In hybrid PET/MR, the two modalities are acquired simultaneously, which avoids image misregistration. The main difficulty is registering the subjects’ images with a referenced brain atlas. Many studies have focused on developing or optimizing algorithm performance for cortical structures in support of the functional MRI literature [22, 23]. However, the evaluation of effects of different automatic segmentation methods on PET SUV quantification is lacking. To label brain regions for group analysis, automatic segmentation RSIAT always fits the subjects’ images to a common reference space [24, 25], and PET quantification is performed in the transformed space, which fits the individual image data to adapt to the common reference frame and probably influences the accuracy of regional SUVs [26].

## Materials and methods

In this study, we inversely registered the atlas in the template space to the original PET/MR data space and calculated the SUV—defined as registering atlas template to subjects’ images (RATSI) method—then compared the quantification with that of the traditional RSIAT method of fitting the clinical image data to the template space. Using the two automatic methods, we segmented two representative brain areas containing four regions: the left caudate (CAU_L), right caudate (CAU_R), left putamen (PUT_L), and right putamen (PUT_R) in twenty PD patients, then compared the SUV_mean_ and SUV_max_ in the corresponding brain regions. The manual segmentation method was also performed and used as the ground truth. For quantitatively evaluating the two atlas-based automatic methods, the segmented results from the three methods (including the manual method) were normalized into the same MNI space in the end; then, the Dice coefficient (DC) and Hausdorff distance (HD) were calculated to evaluate inter-rater variability. The RATSI method was applied to quantify the differences in ^18^F-FDG uptake between PD and MSA groups in multiple brain regions, including caudate, putamen, and the cerebellar gray matter.

### Subjects and data

We retrospectively studied patients who had undergone ^18^F-FDG PET/MR brain examinations for diagnosing or evaluating neurodegenerative diseases in our PET center (Wuhan Union Hospital, Wuhan, China). The study was approved by the Ethics Committee of Tongji Medical College, Huazhong University of Science and Technology. Patients provided written informed consent.

Twenty typical PD (60 ± 5 years) and eight MSA patients (60 ± 8 years) were involved in this study. The diagnosis was according to the diagnostic criteria for PD in China in 2016 and the MSA diagnostic criteria of a Chinese expert consensus in 2017. The exclusion criteria were as follows: (1) a clear history of stroke, with brain MRI examination revealing large cortical infarction or hemorrhagic manifestations; (2) CNS infectious disease; (3) brain tumors or history of head trauma; (4) history of craniocerebral surgery; and (5) suboptimal image quality.

### Image acquisition and reconstruction

All patients underwent ^18^FDG-PET and MRI brain imaging simultaneously in a hybrid PET/MR scanner (3.0 T, SIGNA TOF-PET/MR, GE Healthcare). The ^18^F-FDG was produced in our center by a Minitrace cyclotron (GE Healthcare, USA) and automatic synthesizer (PAT Biotechnology Company, Beijing, China). The radiochemical purity was > 95%.

All participants fasted for at least 6 h and stopped any drugs that could affect brain glucose metabolism for at least 12 h before the ^18^F-FDG injection. The intravenously injected dose was 0.1 mCi/kg (3.7 MBq/kg) after ensuring the blood glucose level was ≤ 200 mg/dL. The scan began 40 min post ^18^F-FDG injection, during which the subject rested in a quiet and dimly lit room. The total scanning time for PET was 15 min, and the 3D T_1_WI (three-dimensional gradient echo sequence, flip angle = 12°, time of echo [TE]/time of repetition [TR] = 2.6/6.9 ms, bandwidth = 50 KHz, FOV = 24 cm × 24 cm, matrix = 384 × 384) sequence was simultaneously acquired.

The PET data were reconstructed using the ordered subsets expectation maximum (OSEM) algorithm with TOF technique. The parameters were as follows: FOV = 30 cm × 30 cm, matrix = 192 × 192, filter cutoff = 3.0 mm, subsets = 28, iterations = 3. The PET attenuation correction was atlas-based MRI attenuation correction, combined with Dixon water-fat separation methods [27].

### Brain segmentation and SUV quantification

**Fig. 1:**
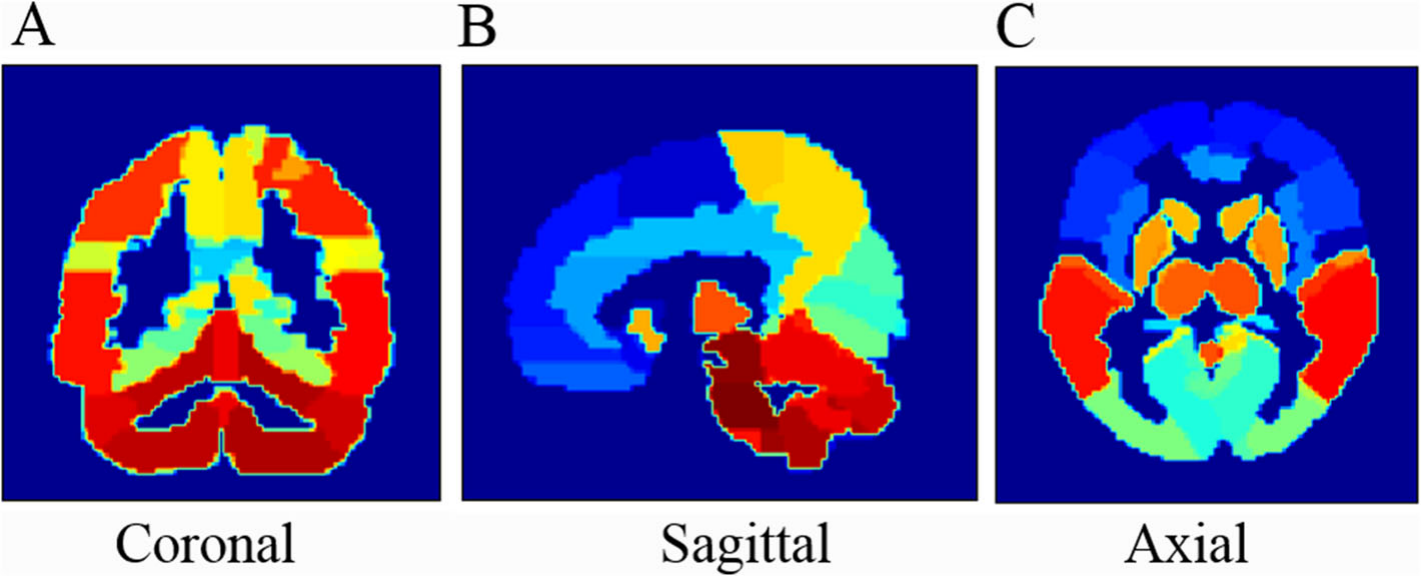
The brain atlas template, which segments the brain into 70 regions, labeled with numbers from 1 to 70 and showed with different colors. It was used for the following automatic segmentations

**Fig. 2:**
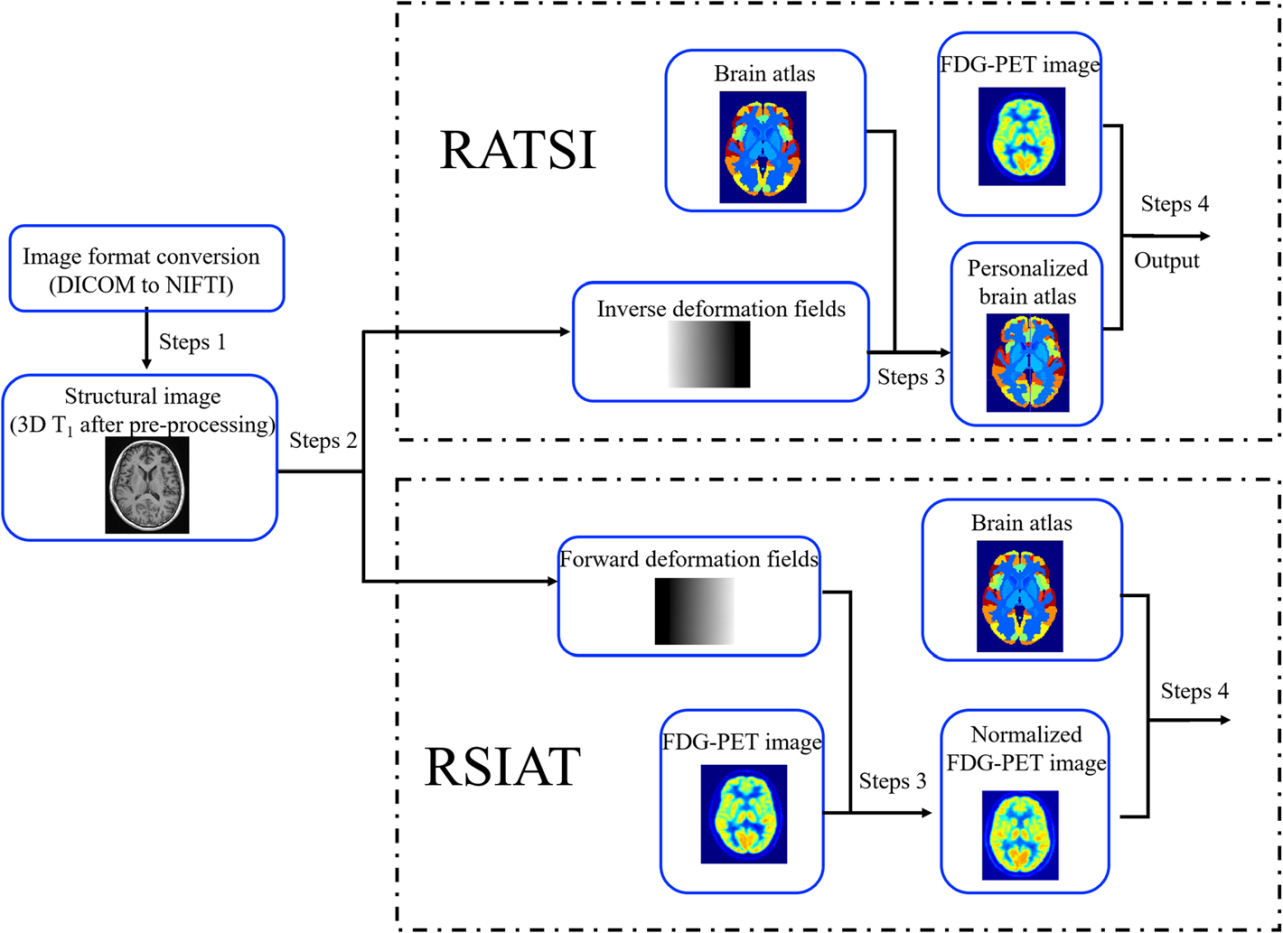
The diagram displaying the processing steps of the two atlas-based automatic methods for whole brain automatic segmentation and regional ^18^F-FDG PET quantification

Automatic brain segmentation was based on an atlas template from the automated anatomical labeling atlas (http://www.gin.cnrs.fr/en/tools/aal-aal2/) shown in Fig. 1. There are 70 segmented regions labeled from 1 to 70 in this brain atlas, which were used for both the two atlas-based automatic methods. By registration of 3D T_1_-weighted MRI to MNI space with SPM12 segmentation (http://www.fil.ion.ucl.ac.uk/spm/download/), the forward and inverse deformation fields could be produced. The RSIAT spatially fitted the ^18^F-FDG PET images to the atlas template with the forward deformation field directly and produced the PET images in the MNI space, which could be segmented directly with the brain atlas. In contrast, the RATSI fitted the acquired inverse deformation field to the brain atlas template, generating a personalized brain atlas for every subject, which then was used for regional ^18^F-FDG PET image quantification, as shown in Fig. 2.

For the two automatic brain segmentations, the detailed steps were as follows:

1. The medicine (DICOM) format of 3D T_1_ MRI and ^18^F-FDG PET images was converted to the neuroimaging informatics technology initiative (NIfTI) format using SPM12 for following processing.
2. The 3D T_1_ images were normalized to MNI space. The results would produce the inverse deformation field (for RATSI) and forward deformation field (for RSIAT), which extracted the information of transformation between the data acquisition space and MNI space.
3. For RATSI, by utilizing the inverse deformation filed, the anatomical labeling atlas in the MNI space was transformed into the data acquisition space and produced the personalized atlas template, which was in the same space with FDG images. For RSIAT, by utilizing the forward deformation field, the FDG-PET images could be normalized to the MNI space.
4. For RATSI, the regional FDG images were segmented according to the personalized atlas template, and further, the mean or max SUV values in different regional brain could be calculated. For RSIAT, the normalized FDG images were registered with the atlas template, then were segmented directly according to the regions-labeled brain atlas. At last, the regional SUV values could be calculated.

Manual segmentation was performed by a clinical neuroimaging expert using ITK-SNAP (http://www.itksnap.org) section by section, using the 3D T_1_ structural images. As the manual method was time-consuming, only two cerebral nuclei containing four regions (left caudate, right caudate, left putamen, and right putamen) were extracted and used for evaluation of the automatic segmented results. The extracted regions based on structural images produced the corresponding binary mask, which used for ^18^F-FDG PET images segmentation.

The regional SUV calculations were performed with Matlab 2016a (Mathworks, Natick, MA, USA). The SUVs were calculated by [28]

1 $$ \mathrm{SUV}=\frac{r}{a^{\prime }/w}, $$

where *r* is the radioactivity concentration [kBq/mL], *a*^′^ is the decay-corrected amount of injected radiolabeled ^18^F-FDG [kBq], and *w* is the weight of the patient [g].

### Inter-rater reliability

The four brain regions were segmented with the manual method on twenty PD subjects for inter-rater variability evaluation by using the parameters DC and HD. The DC evaluates the similarity between two volumes by measuring their overlap [29].

2 $$ \mathrm{DC}=\frac{2\left|A\bigcap B\ \right|}{\left|A\ \right|+\left|B\ \right|}, $$

where *A* and *B* represent the segmentation volumes of the automatic methods and manual method, respectively. *A*∩*B* represents the intersection of the two volumes. A DC value of 1 represents two identical segmentations while a DC value of 0 represents no overlap between the two segmentations. HD usually measures how far two subsets of a metric space are from each other, and here, determines on average how much the two segmented volumes differ. A smaller HD represents a closer agreement between two volumes.

### Statistical analysis

The differences in parameters DC and HD were analyzed by a paired *t*-test. The four segmented brain regions used as binary masks were overlapped on the PET images to extract the regional SUV_mean_ and SUV_max_. One-way analysis of variance (ANOVA) was used to compare the differences in quantitative SUVs among the three segmentation methods. The *F*-test was used to test whether the variance was homogeneous, and the two-tailed *t*-test was used to compare the differences in SUVs in the different regions in the basal ganglia and cerebellar gray matter between the PD group and MSA group. *P* > 0.05 was considered variance homogeneous for *F*-test. *P <* 0.05 was considered statistically significant for *t*-test.

## Results

**Fig. 3:**
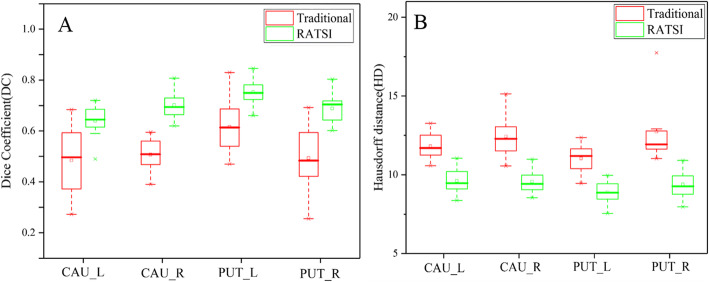
The boxplots displaying distributions of the Dice coefficient (**a**) and Hausdorff distance (**b**), which were used to evaluate the brain segmentation accuracy in compared with the ground truth, the manual segmentation results. The red and green boxplots represented the results of RSIAT method and RATSI, respectively. Representative nuclei including caudate nucleus (left: CAU_L, right: CAU_R) and putamen (left: PUT_L, right: PUT_R) were segmented for analysis

The DC and HD for the RSIAT method (green boxplots) and RATSI method (red boxplots) are displayed in Fig. 3. The mean DCs of RATSI were much larger than those of the RSIAT method, while the mean HD of RATSI was much smaller. The quantitative values are listed in Table 1. The corresponding two-tailed *t*-test results are also shown. Significant differences in DC and HD were found between the two methods (*P* < 0.05). The maximum DC was nearly 0.8 for right caudate nucleus segmentation in the RATSI method.

**Table 1:** The Dice coefficient (DC) and Hausdorff distance (HD) (mean ± SD) for evaluating the accuracy of automatic brain segmentation quantitatively

			^**1**^CAU_L	^**2**^CAU_R	^**3**^PUT_L	^**4**^PUT_R
**DC**	Methods	Traditional	0.48 ± 0.13	0.51 ± 0.06	0.62 ± 0.09	0.49 ± 0.12
		RATSI	0.64 ± 0.06	0.70 ± 0.05	0.75 ± 0.05	0.69 ± 0.05
	***P*< 0.001**					
**DH (pixels)**	Methods	Traditional	11.80 ± 0.74	12.42 ± 1.19	11.03 ± 0.88	12.73 ± 1.87
		RATSI	9.61 ± 0.75	9.56 ± 0.69	8.89 ± 0.68	9.39 ± 0.75
	***P*< 0.001**					

^1^CAU_L: left caudate nucleus

^2^CAU_R: right caudate nucleus

^3^PUT_L: left putamen

^4^PUT_R: right putamen

**Fig. 4:**
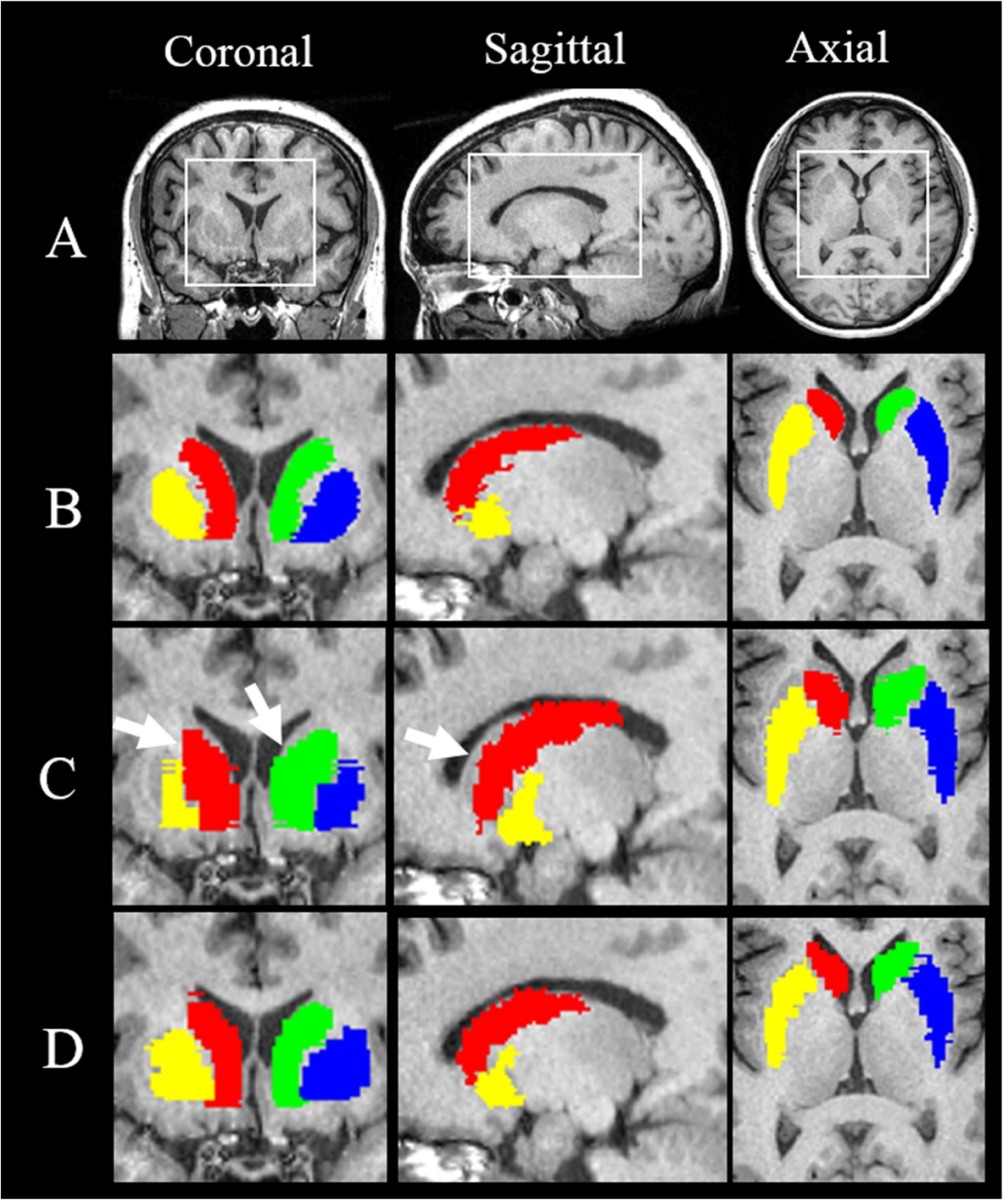
Representative visualization of segmented brain nuclei including caudate nucleus (green: CAU_L, red: CAU_R) and putamen (blue: PUT_L, yellow: PUT_R) by using manual segmentation (**b**), RSIAT method (**c**), and RATSI (**d**). The areas in the white box in T_1_ images (**a**) were the regions shown below. And the segmented regions of interest (ROIs) for nuclei were overlapped on T_1_ images in coronal (left), sagittal (middle), and axial views (right), respectively, for better visualization

Figure 4 shows the representative segmented caudate and putamen ROIs on the left and right side with the three segmentation methods, which were overlain on T_1_WI and displayed with different colors for better visualization, including coronal, sagittal, and axial views. Visually, the segmented volumes from the RSIAT method were larger than the manual and RATSI segmentation volumes, especially for the right caudate nucleus as indicated by the white arrows.

**Fig. 5:**
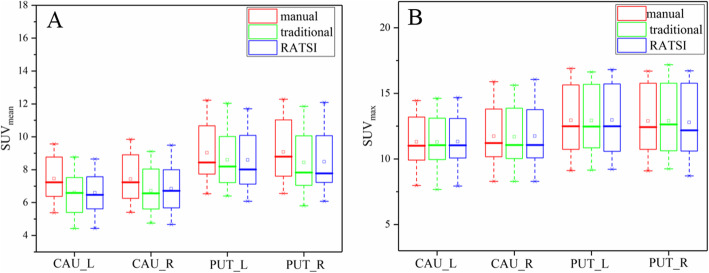
The boxplots displaying the distribution of the SUV_mean_ (**a**) and SUV_max_ (**b**) in caudate nucleus (left: CAU_L, right: CAU_R) and putamen (left: PUT_L, right: PUT_R) from twenty PD patients with manual segmentation (red), the RSIAT method (green), and RATSI (blue)

The distributions of SUV_mean_ and SUV_max_ extracted with the three segmentation methods in the caudates and putamina from all twenty PD patients are box-plotted in Fig. 5. For SUV_max_, the quantification was nearly the same, while the SUV_mean_ of both the automatic segmentation methods was slightly lower than those of the manual segmentation. The corresponding quantitative SUV and ANOVA results are listed in Table 2. No significant differences were found in SUV_mean_ or SUV_max_ among the three segmentation methods (*P* > 0.05).

**Table 2:** The lists of mean and max ^18^F-FDG-SUVs in segmented regions extracted with manual segmentation, RSIAT, and RATSI

			CAU_L	CAU_R	PUT_L	PUT_R
**SUV_mean**	^1^Manual segmentation		7.45 ± 1.39	7.42 ± 1.42	9.03 ± 1.69	9.08 ± 1.76
	^2^Traditional		6.61 ± 1.38	6.72 ± 1.37	8.60 ± 1.63	8.44 ± 1.71
	^3^RATSI		6.60 ± 1.37	6.84 ± 1.36	8.59 ± 1.72	8.48 ± 1.67
	*P* (among groups)		*0.090*	*0.233*	*0.642*	*0.419*
	*P*	1 vs 2	*0.059*	*0.111*	*0.419*	*0.239*
		1 vs 3	*0.055*	*0.187*	*0.415*	*0.272*
		2 vs 3	*0.974*	*0.778*	*0.995*	*0.937*
**SUV_max**	Manual segmentation		11.29 ± 2.04	11.73 ± 2.32	12.95 ± 2.47	12.90 ± 2.59
	Traditional		11.29 ± 2.12	11.68 ± 2.38	12.92 ± 2.44	12.90 ± 2.63
	RATSI		11.32 ± 1.93	11.74 ± 2.32	12.97 ± 2.47	12.78 ± 2.66
	*P*		> *0.90*			

**Fig. 6:**
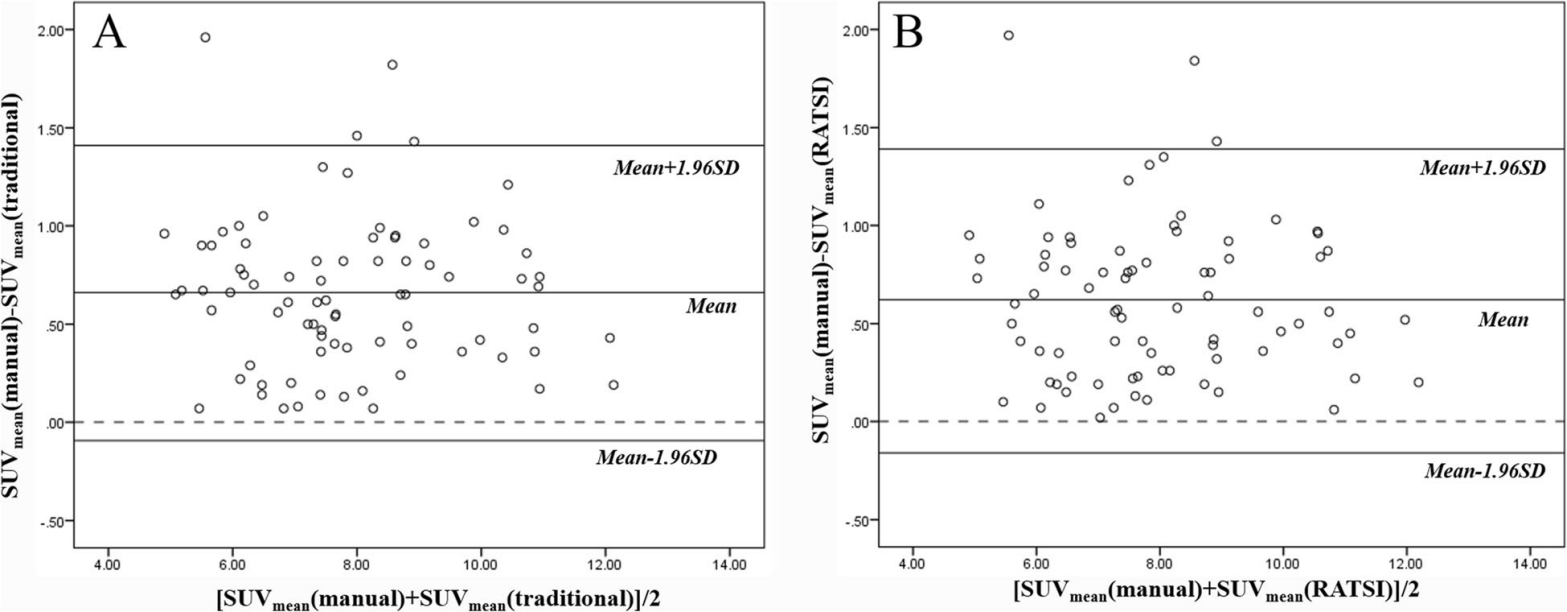
Bland–Altman graphs to evaluate the SUV_mean_ consistency of the RSIAT method (**a**) and RATSI (**b**) in comparison with the manual segmentation in the four representative regions including the left, right caudate nucleus, and putamen. The SD represents the standard deviation

The consistencies of SUV_mean_ obtained from manual and automatic methods were evaluated with Bland-Altman plots. As shown in Fig. 6, the transverse and longitudinal axis represents, respectively, the mean and differential values calculated by the two automatic methods. Most (94%) of the dots were within the two 95% consistency limit lines, which indicated that it is feasible to measure the SUV_mean_ with the automatic segmentation method based on the atlas template.

The SUV_mean_ extracted with RATSI in brain nuclei between the PD group and MSA group is summarized in Table 3. All the SUV_mean_ were decreased in the MSA group compared with those in the PD group. The difference in SUV_mean_ in the cerebellar gray matter was statistically significant between the two groups (*P* < 0.05).

**Table 3:** The SUV_mean_ quantification in brain nuclei for PD and MSA patients

		CAU_L	CAU_R	PUT_L	PUT_R	^**1**^CGM_L	^**2**^CGM_R
**SUV_mean**	PD	6.60 ± 1.37	6.84 ± 1.36	8.59 ± 1.72	8.48 ± 1.67	5.49 ± 0.70	5.32 ± 0.79
	MSA	5.46 ± 1.78	5.87 ± 1.77	7.27 ± 1.81	7.32 ± 1.83	3.93 ± 1.05	3.59 ± 1.06
	***F***	0.50	0.39	0.80	0.83	0.19	0.34
	^3^***P***	***0.07***	***0.21***	***0.15***	***0.15***	***0.00***	***0.00***

^1^CGM_L: left cerebral gray matter

^2^CGM_R: right cerebral gray matter

^3^*P* < 0.05 was considered statistically significantly different for comparisons

## Discussion

The quantitative analysis of regional cerebral metabolism is very meaningful for diagnosis of nervous system diseases and exploring brain function. In this study, by using hybrid PET/MR, the registration of PET functional images and MRI structural images was avoided. Furthermore, the personalized templates, which were finally used for ^18^F-FDG PET regional segmentation and quantification, were built based on an inverse deformation field obtained by registering the atlas template to 3D T_1_WI in SPM12. Compared with the RSIAT method, the RATSI could acquire larger DCs, more consistent with those of the manual method. For the RSIAT, it was the registration process to normalize the PET/MR images to fit the atlas template, which probably change the personalized images, especially for some subjects whose brain structure have changed. In contrast, the RATSI normalized the atlas template to fit the PET/MR images, for producing the personalized atlas, which could avoid the problem from the RSIAT. Therefore, the RATSI registration method is probably advantageous for PET quantitative analysis, especially for point-to-point image analysis. In addition to ^18^F-FDG PET, this method could also be used for cerebral PET analysis with other tracers.

The ability to distinguish between PD and Parkinson syndrome is very difficult, especially in the early stage. Previous reports suggested the diagnostic accuracy rate of early PD was only 53% [30]. In our study, the RATSI method was used for calculation and comparisons of regional brain SUVs between the PD and MSA groups. The results suggested the method can effectively quantify the regional SUVs and find the differences of ^18^F-FDG PET metabolic pattern between the PD and MSA groups. The SUV_mean_ was significantly lower in the cerebellar gray matter in the MSA group compared with the PD group. The results are consistent with previous clinical studies and meta-analyses that use the metabolic brain network based on ^18^F-FDG-PET for the differential diagnosis of Parkinson syndrome [31, 32].

The quantitative evaluation of segmentation accuracy with DC and HD suggested the RATSI can generate better segmented results than the RSIAT method. However, the SUV_max_ was not significantly different, due to that the SUV_max_ usually was less related with the edge of segmented regions. Also, there were no significant differences in the SUV_mean_ among the three segmentation methods in the caudate and putamen. We speculate that because the caudate and putamen are relatively large and contain so many voxels; the marginal differences caused by the two automatic segmentation methods had little influence on the SUV_mean_. Therefore, the RATSI method probably would be more advantageous for smaller areas.

There are still some limits in the study. Firstly, the personalized atlas template was very important for segmentation in the RATSI method, which depended on the deformation field. In this study, for generating the deformation field consistently, the default parameters were used on the SPM12. The parameters for every registration should be optimized to acquire more precise and personalized deformation field data. Secondly, besides the SPM12, lots of other software could be used for realizing the two segmented methods, i.e., FSL or FreeSurfer. The results of the comparison from other software were unknown. Ewert et al. practically optimized parameters and compared subcortical alignment for the following nonlinear image registration tools: Normalization Tools ANTs 2.2.0, SPM12 software, and FSL 5.0.10 for atlas-based segmentation of DBS target nuclei [17]. The corresponding process could be referenced in the future for more detailed research. Thirdly, partial volume effect is a common phenomenon in the medical imaging equipment, especially for PET images due to the relatively low spatial space. In this study, we did not perform the partial volume effect correction in consideration that the two segmentation methods were mainly based on 3D T_1_ MRI. However, the partial volume effect may reduce the accuracy of PET quantitative analysis and have some influence for comparing regional differences in cerebral metabolism pattern between PD and MSA patients. Therefore, partial volume effect should be noted in the future study.

In this study, we focused on manual and automatic segmentation and quantification evaluation of the caudate and putamen. The automatic method could be theoretically extended to other structures according to the atlas templates. However, some deep brain structures show poor contrast and are typically difficult to be visualized on T_1_WI, e.g., the red nucleus and substantia nigra, but are better visualized on T_2_WI. Fonov et al. [33] and Xiao et al. [34] demonstrated better segmentation results from non-rigidly warping the T_1_WI and T_2_WI to a common template space. In the future, multi-modality data, that is, T_1_WI, T_2_WI, and PDWI, might be used for automatic segmentation. This needs to be explored in the future.

## Conclusions

We utilized two automatic segmentation methods for regional PET analysis. Comparing with the traditional RSIAT method, the RATSI was more accurate for the caudate nucleus and putamen automatic segmentation, while has little effects for their max and mean SUV calculation in hybrid PET/MR. And it could be theoretically extended to other structures according to the atlas templates. Further, the regional PET results from the RATSI method have been demonstrated useful for the differential diagnosis between the PD and MSA.

## Data Availability

The datasets used and/or analyzed during the current study are available from the corresponding author on reasonable request.

## Abbreviations

PET: Positron-emission tomography
RATSI: Registering atlas template to subjects’ images
RSIAT: Registering subjects’ images to atlas template
PD: Parkinson disease
MSA: Multiple system atrophy
PET/MR: Positron-emission tomography/magnetic resonance imaging
DC: Dice coefficient
HD: Hausdorff distance
SUV: Standardized uptake value
ANOVA: One-way analysis of variance
CT: Computed tomography
MNI: Montreal Neurological Institute
